# A novel drug repurposing approach for non-small cell lung cancer using deep learning

**DOI:** 10.1371/journal.pone.0233112

**Published:** 2020-06-11

**Authors:** Bingrui Li, Chan Dai, Lijun Wang, Hailong Deng, Yingying Li, Zheng Guan, Haihong Ni

**Affiliations:** Beijing Deep Intelligent Pharma Technologies Co., Ltd, Beijing, China; Biotechnology HPC Software Applications Institute (BHSAI), UNITED STATES

## Abstract

Drug repurposing is an attractive and pragmatic way offering reduced risks and development time in the complicated process of drug discovery. In the past, drug repurposing has been largely accidental and serendipitous. The most successful examples so far have not involved a systematic approach. Nowadays, remarkable advances in drugs, diseases and bioinformatic knowledge are offering great opportunities for designing novel drug repurposing approach through comprehensive understanding of drug information. In this study, we introduced a novel drug repurposing approach based on transcriptomic data and chemical structures using deep learning. One strong candidate for repurposing has been identified. Pimozide is an anti-dyskinesia agent that is used for the suppression of motor and phonic tics in patients with Tourette’s Disorder. However, our pipeline proposed it as a strong candidate for treating non-small cell lung cancer. The cytotoxicity of pimozide against A549 cell lines has been validated.

## Introduction

Although the knowledge and technology of human diseases have developed substantially, the translation of these benefits into therapeutic innovations has been far slower than expected [1, 2]. The main challenges facing global pharmaceutical industries are substantial costs and long consuming time in the process of drug discovery and development [2]. To solve this problem, drug repurposing (also known as drug repositioning, re-tasking or reprofiling) has emerged as an attractive and pragmatic way offering reduced risks and development time [3]. In the past, drug repurposing has been largely accidental and serendipitous. The most successful examples so far have not involved a systematic approach. Nowadays, the boom of drugs, diseases and bioinformatic knowledge is offering great opportunities for designing novel drug repurposing approach [4–8].

On one hand, a number of approaches try to identify an analogue of an existing drug molecule sharing mechanisms of action with the original drug [2, 9, 10]. It means that drugs belong to different therapeutic use classes with similar chemical structures, are most likely to have same indications. This principle can find alternative targets of existing drugs and uncover potential off-target effects that can be investigated for clinical applications [11]. On the other hand, regardless of the similarity in drug structures, a similar transcriptomic signature between two drugs may share the same indications [2]. These approaches make use of gene expression data before and after drug perturbations to construct a network of transcriptional response and functional properties of drugs [12–14]. Ideally a drug may have the potential to cure a disease if the differential expression profile under drug perturbations is anti-correlated significantly [15].

In this study, we leverage both chemical structures and transcriptomic data for repurposing drugs. The potential candidate drugs identified with both chemical structural and transcriptomic signatures, are more likely to successfully progress through the drug discovery lifecycle. The approach includes the classifying process and the repurposing process. The former relies on a transcriptomic database, based on deep neural networks (DNNs) that is trained on large sets of perturbation samples of X drugs from the Library of Integrated Network-Based Cellular Signatures (LINCS) and links those to 6 therapeutic use classification derived from Medical Subject Headings (MeSH) therapeutic use categories. Based on transcriptional profiles as drug classification, we predict misclassified antineoplastic drugs which belong to another therapeutic use categories in MeSH. Then by chemical structure similarity comparison with non-small cell lung cancer (NSCLC) drugs approved by U.S. Food & Drug Administration (FDA), we obtain a list of potential drugs ranked by the estimation of both pathway activation score and structure similarity score. The proposed candidates for NSCLC are validated experimentally in the lab and discussed in the results. The approach established in this article can be extended to other diseases, holding great promise for shortening the drug discovery process.

## Materials and methods

### Drug data

MeSH classification (https://www.nlm.nih.gov/mesh/) was utilized to symbolize drugs profiled from LINCS. Only the drugs with the linkage to one disease in “therapeutic use” section were chosen in this study. We used the perturbation samples of 75 drugs and linked them to 6 therapeutic use categories derived from MeSH. The 6 therapeutic use categories include vasodilator agents, anti-dyskinesia agents, anticonvulsants, hypolipidemic agent, anti-asthmatic agent, and antineoplastic agents.

### Gene data

In this work, the gene expression data was obtained from the LINCS Project participants (http://www.lincsproject.org/) which was downloaded from NCBI Gene Expression Omnibus (GEO,GSE70138 (https://www.ncbi.nlm.nih.gov/geo/query/acc.cgi?acc=GSE70138)). LINCS is a National Institutes of Health (NIH) program which provides gene-expression profiles across multiple cells and perturbation types, as well as read-outs, at a massive scale. The level 3 (Q2NORM) gene expression of three cell lines: A375, HELA and HT29 were used. (GSE70138_Broad_LINCS_Level3_INF_mlr12k_n345976x12328_2017-03-06.gctx.gz.) The level 3 (Q2NORM) are gene expression profiles of both directly measured landmark transcripts and inferred gene. Landmark genes were normalized by invariant set scaling followed by quantile normalization. The final gene expression dataset has 12,797 genes.

### Signaling pathways and topological weight

We obtained signaling pathways from Kyoto Encyclopedia of Gene Genomics (KEGG) (https://www.kegg.jp/kegg/pathway.html). A signaling pathway in KEGG is graphed with nodes and directed edges representing genes or proteins, and signal relationships, respectively. The edges are weighted according to activation and inhibition. In this study, we obtained a set of 164 signaling pathways covering 4414 unique genes. The contributions of genes were multiplied by an integer which equals to +1/-1 and +2/-2 in the case of pathway activation/suppression and phosphorylation/dephosphorylation, respectively. KEGG graph (http://www.bioconductor.org/packages/release/bioc/html/KEGGgraph.html) and Boost Graph Library (BGL) R packages [16, 17] were downloaded from Bioconductor (https://www.bioconductor.org/packages/release/bioc/html/RBGL.html). Signaling pathway xml files from KEGG were used as the input for the calculation of topological weight.

### Grouping genes into modules

Human database of coexpressed genes COXPRESdb [18] was used in order to obtain the gene modules. We used Euclidean distance matrix to cluster correlation data from COXPRESdb. We used the following equation to calculate distances:

$$R_{ij}=1-corr_{ij}$$

Where corr_ij_ is the correlation between expression levels of genes i and j. Ordering Points To Identify the Clustering Structure (OPTICS) [19] was firstly used to identify clusters. Clusters with the average internal pairwise correlation below 0.5 were excluded in our study. Then we merged all the clusters from OPTICS into a group and applied Balanced Iterative Reducing Clustering using Hierarchies (BIRCH) [20] to cluster the group again. Finally, a data set of 177 gene modules including 1271 unique genes was built.

### Pathway score matrix

The pathway score matrixes were calculated based on *in silico* pathway activation network decomposition analysis (iPANDA) algorithm [21] with coexpressed genes. The input files were KEGG signaling pathway topological weights and gene list. The output file was an activation score matrix with 3985 samples in 164 signaling pathways.

### Classifying process

We selected all samples corresponding to different perturbation concentration, time and cell lines. “Landmark genes” and signaling pathways were new features for training our models. So-called “landmark genes” were derived from the LINCS project. For classification methods, we chose two robust and widely used methods including random forest (RF) and deep neural network (DNN) methods. The feature of RF is that it can build a large number of regression trees and average their predictions, allowing for high dimensional flexible modeling of interactions. Nevertheless, these parameters are not evident for a given data set [22]. Grid search was utilized for hyperparameter optimization. The RF has been trained via nested 3-fold cross validation, the first 66.7% columns of the full data matrix were selected as a training set, while the remaining 33.3% of the columns were reserved as the test set. Using 400 trees and default values for other parameters, the trained model was subsequently used to predict the class of the drugs for test datasets. In this study, DNN has fully connected multilayer perception with 978 input nodes for gene expression and 163 input nodes for signaling pathways. The DNN model was trained via 10-fold cross validation, the first 90% data were served as training set, while the remaining 10% data were reserved as test data. Grid search for hyperparameter optimization of optimal number of layers, nodes of each layer and dropout rejection rate were also utilized in order to compare the performance of RF. The number of layers were trained from 3 to 5, while the nodes in each layer were started from 100 to 400 in steps of 50. Each layer was started by Glorot uniform approach [23]. At each layer, the dropout rejection ratio was 10% and 50%, respectively. Rectified linear unit (ReLU) [24], as nonlinear function of hidden neurons was utilized to speed up the process of the calculation, especially for the large input. The output nodes number was 6, which was the same with our number of classes. Finally, we found the number of 3 hidden layers with 150 in each with rectified linear activation function was the optimal parameter. The source code can be found on github (https://github.com/ai-diper/DNN-DR).

### Repurposing process

Library for the Enumeration of MOdular Natural Structures (LEMONS) is a software package originally designed to enumerate hypothetical modular natural product structures [25]. However, in this study, we utilized LEMONS to compare the structure similarity between two compounds based on Functional-Class Fingerprint (FCFP). Tanimoto coefficient, as one of the best metrics for similarity calculations, was used to compare structure similarities of small molecules. After computing, the drugs from our repurposing drug pool with tanimoto coefficient above than 0.8 were selected for further analysis. The tanimoto coefficient of 0.8 reflected a high probability of two compounds sharing the same activity [26, 27]. Finally, all these potential drugs were ranked according to combinational score of Di-PASS score and chemical structure similarity score.

### *In vitro* experiments

Two NSCLC cell lines (A549 and H157), were used for *in vitro* cytotoxic activity testing using the SRB assay [28]. The rapidly growing cells were harvested, counted, and incubated at the concentration of 1 × 10^4^ cells/well in 96-well plates. After incubation for 48h, the compounds (including pimozide and gemcitabine) were dissolved in culture medium, applied to the culture wells in triplicate, and incubated for 48 h at 37°C in a humidified incubator. The cultured cells were fixed with 50% (m/V) (50 mg/100 ml) trichloroacetic acid at 4 °C for 1 h. Then 0.4% SRB was dissolved in 1% acetic acid for each well for 30 min. 10 mM unbuffered Trisma base solution was used for solubilizing the bound stain with a gyratory shaker. The optical density (OD) of 515 nm was measured spectrophotometrically in a microplate reader. Cytotoxic activity was evaluated by identifying the concentration of compound that was required to inhibit cell growth by 50% (IC_50_) [29]. Each experiment was operated for three times. We used the following equation to calculate IC_50_:

$$IC_{50}=[(OD_{control}-OD_{compound})/OD_{control}]\times 100\%.$$

### Ethics approval and consent to participate

Cells used in this study were obtained from National Infrastructure of Cell Line Resource and used according to the approved protocols.

## Results and discussion

**Fig 1:**
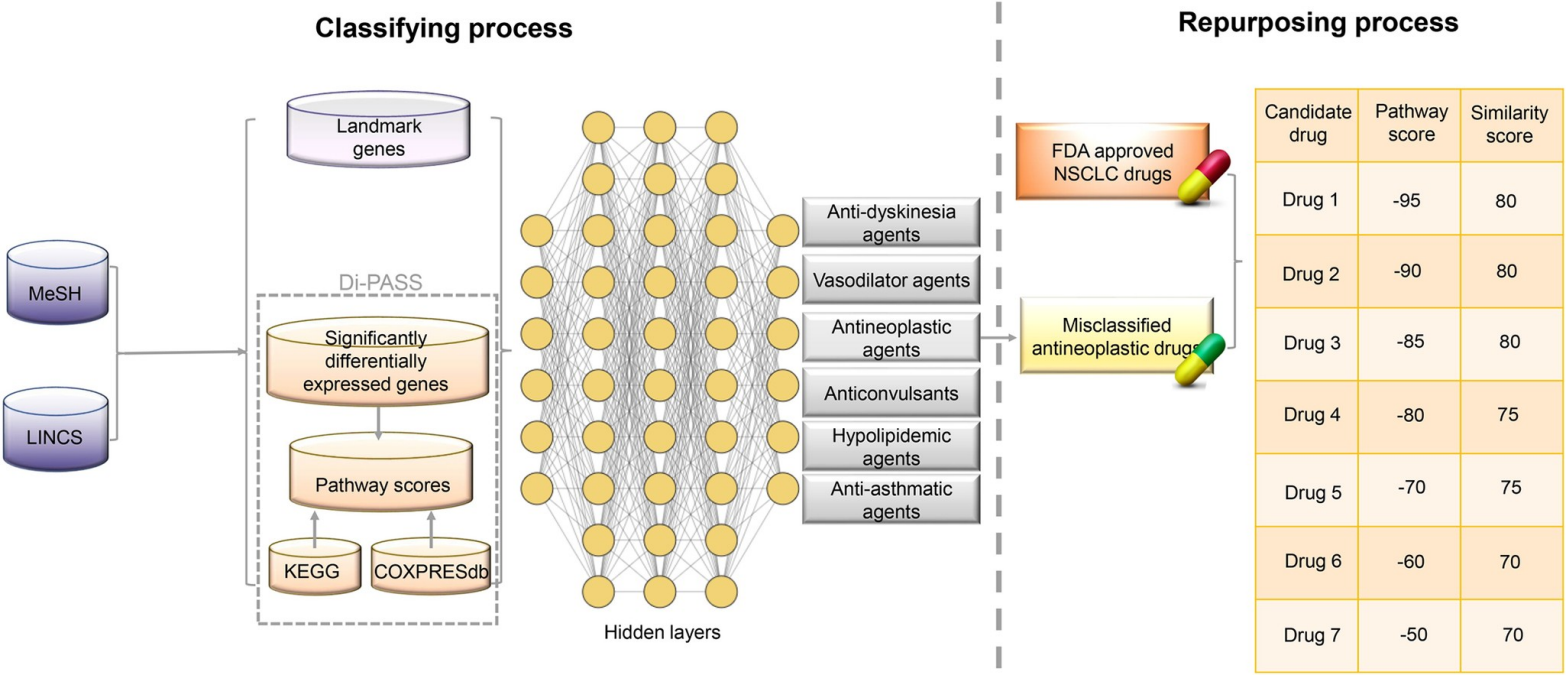
The schematic illustration of study design.

This paper aimed at providing a novel drug repurposing approach to discover alternative NSCLC drugs from existing drugs. In the repurposing process, we not only considered the chemical structural information of molecules, but also leveraged transcriptomic data for discovering new indications (Fig 1). All data including transcriptional data of the reference and perturbation samples were obtained from LINCS. Pathway activation scores were computed by DIP^®^ Pathway Activation Scoring System (Di-PASS) based on differentially expressed gene data, Kyoto Encyclopedia of Genes and Genomes (KEGG), and COXPRESdb. In order to reduce biologically relevant dimensions, landmark genes and signaling pathways were chosen as new features for predicting new therapeutic use categories. Based on the classification using transcriptional data only, several misclassified antineoplastic drugs were obtained, which had different therapeutic roles in MeSH. Through combinational ranking of both chemical structures and corresponding pathway activation scores, new potential drugs were discovered. Misclassified antineoplastic drugs are compared with FDA approved NSCLC drugs.

### Di-PASS as a tool for pathway activation estimation

**Fig 2:**
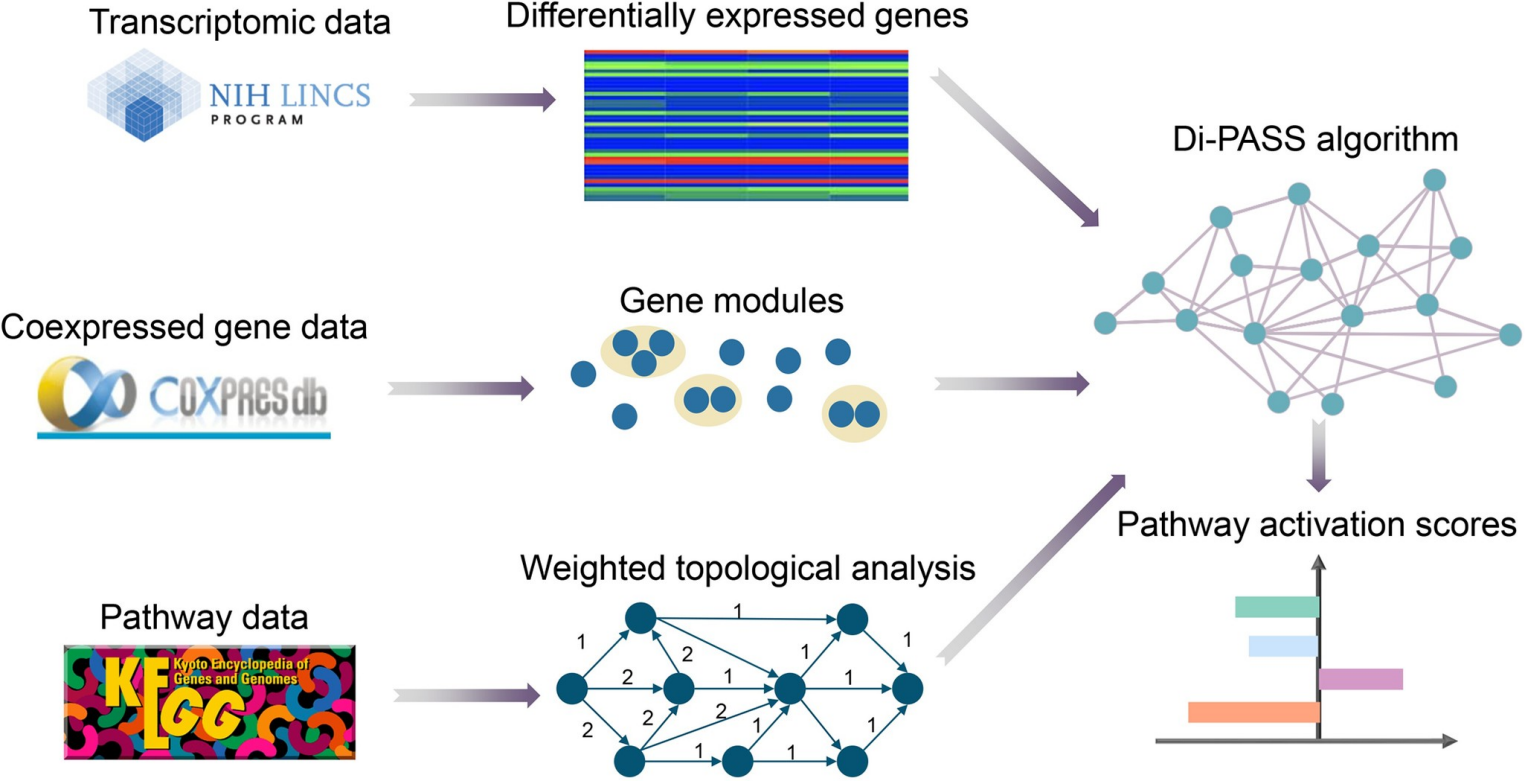
The general scheme of Di-PASS algorithm.

When analyzing and organizing data in high dimensional space, the amount of data required to provide a reliable analysis grows exponentially [30]. This phenomenon is called “curse of dimensionality”, which is a big challenge in the fields of genomics. Common approaches [31,32] only recognize expression signature patterns of the process, thus failing to capture vital differences among samples that come from complex gene network interactions. To circumvent this obstacle, various pathway analysis techniques have been proposed which can integrate gene expression data into molecular signaling networks, thus reducing biologically relevant dimensions [31]. Some widely used pathway-based algorithms, for instance, Gene Set Enrichment Analysis (GSEA), solely focuses on gene enrichment statistics, without structured sets of genes as pathways [32]. There is still an urgent need to invent a novel analytical method which can build an accurate network of biological signaling from complex transcriptomic data. In this study, we introduced Di-PASS algorithm that integrated different analytical concepts such as gene expression data, gene importance factor [33], and topological weights into a single network, simultaneously exploiting Di-PASS scores for pathway activation estimation (Fig 2). The gene expression data was obtained from the LINCS Project participants. The level 3 (Q2NORM) gene expression of three cell lines: A375, HELA and HT29 was used. After drug perturbation, genes with insignificant change expression were excluded from further analysis. It is well known that several genes exhibit closely connections in their expression level, which can be considered as gene coexpression [18, 34, 35]. COXPRESdb (http://coxpresdb.jp) is a database which first release coexpression information for human and mouse. In this work, we used COXPRESdb [18, 36] for moduling, because of its reduced false positive relationships in individual gene coexpression data. In addition, topological weight was introduced in order to give more weight to the genes that occupied the central position on the pathway map. In this study, the topological weight of each gene was set proportional to the number of independent paths through the pathway. Then the computation of topological weight was estimated for each module as a whole rather than for individual genes [21]. The pathway data was obtained from KEGG, which constructed manually curated pathway maps that represent current knowledge on biological networks in graph models [37]. The contribution of gene units (either individual genes or gene modules) to pathway activation was calculated as an output of their fold changes and topological weights. In the end, the output results, referring to pathway activation scores (Di-PASS scores), were produced as signed scores showing the direction and intensity of pathway activation.

The input data for Di-PASS algorithm includes three parts. The first part started from differential expression analysis. After drug perturbation, only significantly differentially expressed genes were selected for further analysis. Coexpressed gene data was obtained from COXPRESdb. Coexpressed genes were grouped into modules, and were given topological weights according to their importance. The pathway data was obtained from KEGG. The contribution of gene units (both gene modules and individual genes) to pathway activation was calculated as a product of their fold changes and topological weights. The contributions were multiplied by a discrete coefficient which equals to +1 or -1 in the case of pathway activation or suppression, and +2 or -2 in the case of phosphorylation or dephosphorylation, respectively. In the end, the output results, referring to pathway activation scores, were produced as signed scores showing the direction and intensity of pathway activation. The source code of Di-PASS is available (https://github.com/ai-diper/Di-PASS).

### Classifying process

When dealing with transcriptional data, we used 3985 drug perturbation samples for 3 cell lines: A375, HELA and HT29 from the Broad LINCS database (S1 Table). All the samples were classified to 6 therapeutic use categories based on MeSH classification of 75 specified drugs (S2 Table). Those drugs belonging to only one category were chosen in our study. In order to solve the problem of “curse of dimensionality [30]”, we utilized landmark genes and signaling pathways as new features to predict drug classifications.

**Fig 3:**
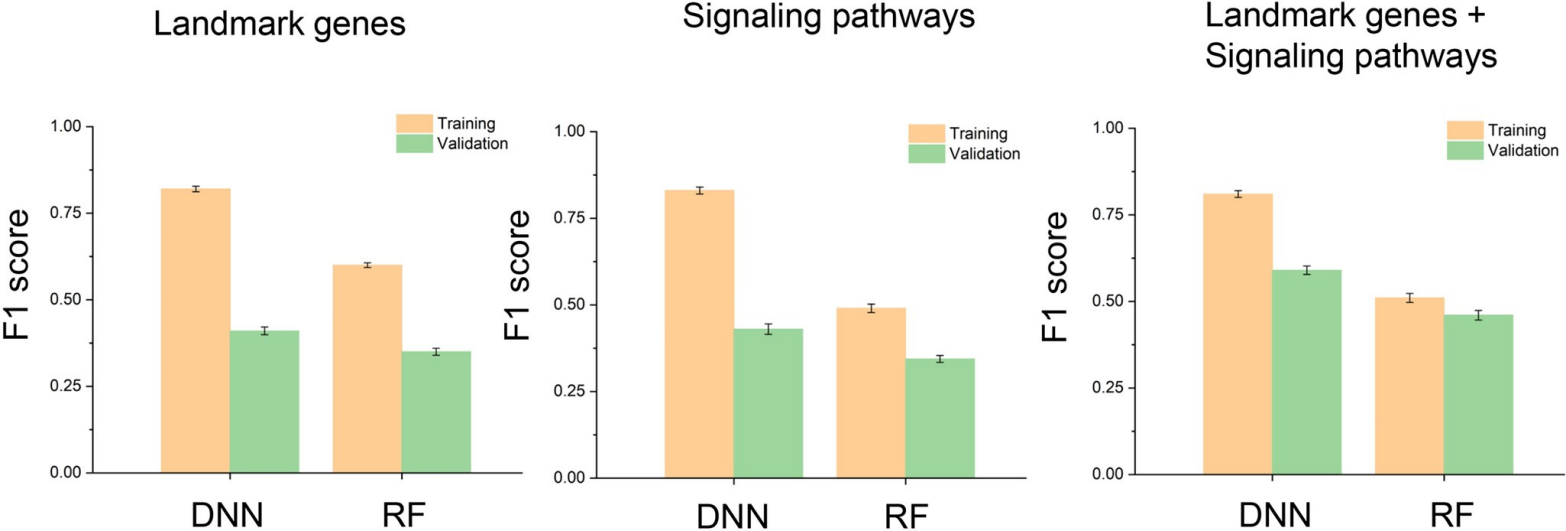
Classification results. Classification performance of DNN and RF trained on landmark gene, signaling pathways, and combinatorial features for 6 drug classes, respectively. Training and validation set were shown in orange and green colors.

For landmark genes analysis, we obtained a data set including normalized gene expression data for 978 landmark genes which captured approximately 80% of the information and possessed great inferential value, according to the authors of LINCS projects [38]. Based on this feature data, we built a classifier using deep learning methods to evaluate the performance of classification on complicated drug action patterns recognition across different therapeutic indications. DNN was the first classifier model we chose. As a high-level representation model of deep learning, DNN outperformed traditional machine learning approaches in the field of automatic task-optimal features learning from deluged data sets [12]. We compared the results from DNN with random forest (RF) via nested 3-fold cross validation for several hyperparameters. Finally, we chose 6 abundant categories: antineoplastic, vasodilator, anti-dyskinesia, anticonvulsants, hypolipidemic, and anti-asthmatic for the class classification. On 6-class classification, DNN and RF performed with mean F1 scores of 0.41 and 0.35, respectively (Fig 3).

For signaling pathways analysis, we used Di-PASS algorithm that performed quantitative estimation of pathway activation intensity. The sign of the Di-PASS scores indicated whether the pathway was activated or suppressed. All the same perturbation samples with those used in landmark genes analysis were estimated with this tool and each sample was computed for 164 signaling pathways (see Methods section for more details). DNN trained on signaling pathway data performed with 10-fold cross-validation mean F1 score of 0.43, while RF performed 0.34 (Fig 3). The results indicated that DNN outperformed RF in classification analysis.

**Fig 4:**
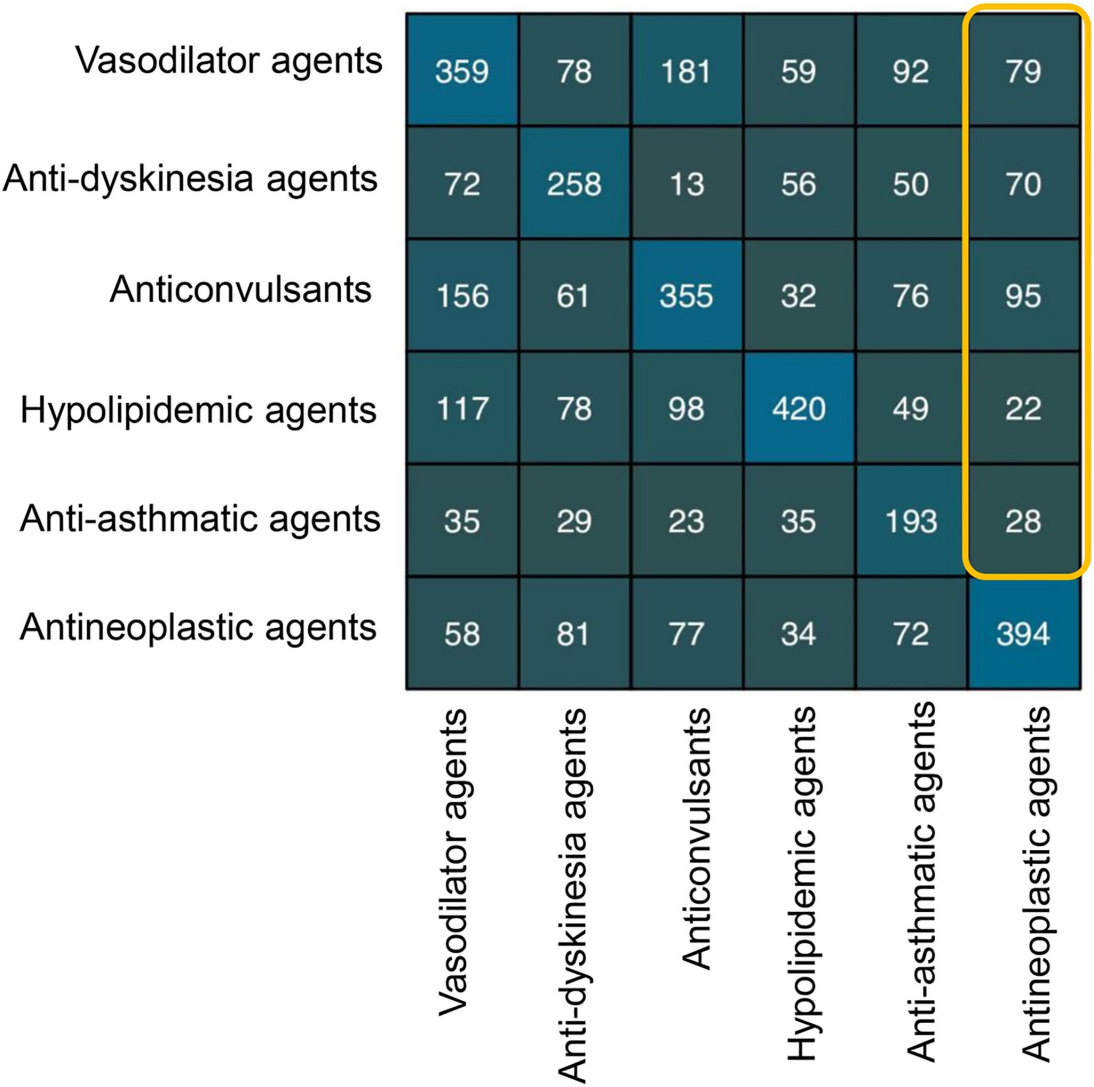
Validation confusion matrix. Validation confusion matrix illustrated DNN classification performance over a set of drugs profiled for A375, HELA and HT29 cell lines, belonging to 6 therapeutic use categories in MeSH. C(i,j) element was the number of samples of how many times i was the truth and j was predicted. The box circled the misclassified false positive drugs.

Considering the low mean F1 score based on the single feature, we combined landmark genes and signaling pathways as new features for classification. DNN performed with mean F1 score of 0.59, while RF performed 0.46 (Fig 3). The results indicated that the combinational features with landmark genes and signaling pathways outperformed the single feature-based methods. DNN was more suitable to classify drugs into therapeutic use categories rather than RF. Thus, DNN with combinational features was selected as the optimal condition for therapeutic use prediction. Finally, we illustrated separability of therapeutic use categories by confusion matrix (Fig 4) utilizing DNN model with landmark genes and signaling pathways as combinational features. Here we observed that vasodilator agents were relatively often misclassified as anticonvulsants and antineoplastic agents. However, the imperfect accuracy here may not be a bad thing. These misclassified false positive drugs are likely to represent a possibility for drug repurposing. For example, nitroglycerin (NTG), as vasodilator agents according to the MeSH therapeutic use section, was discovered to be an antineoplastic agent for its capability of reducing hypoxia-inducible factor (HIF)-1α levels in hypoxic tumor tissues [39]. Another misclassified example is vasodilator agent propranolol, which displays remarkable anti-cancer effects on cellular proliferation and invasion [40]. Here we chose misclassified antineoplastic agents as potential drugs for new indication of treating NSCLC (S3 Table).

### Repurposing process

**Fig 5:**
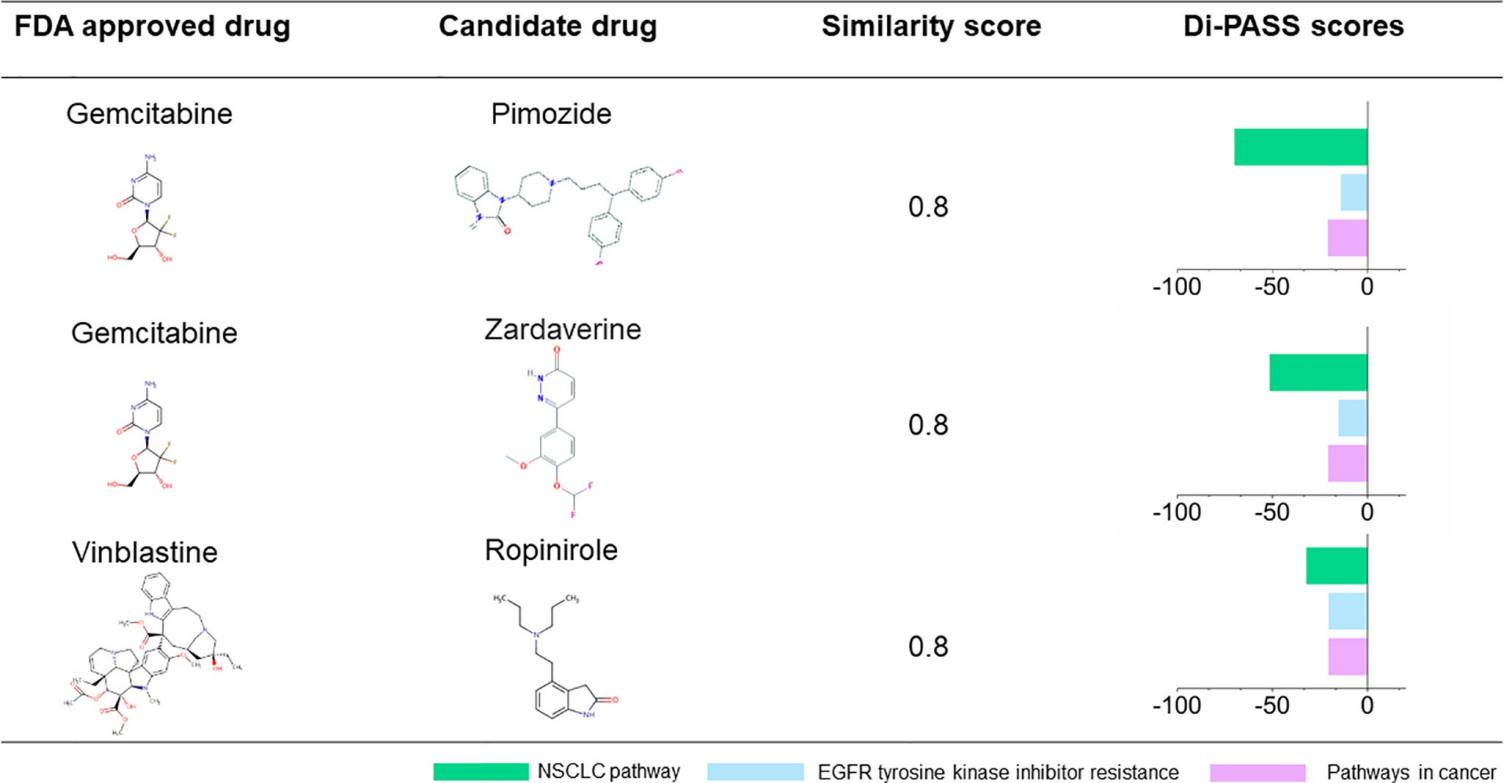
Top candidate drugs ranked by similarity score and Di-PASS scores. Tanimoto coefficient was used for measuring similarity of chemical structures. Di-PASS scores represented the intensity of activation/depression of drugs in NSCLC related pathways.

The commonly used drug repurposing approach is the analogue based approach that aims to identify an analogue of an existing drug molecule sharing mechanisms of action with the original drug [2, 9]. The similarity of chemical structure may suggest shared biological or physicochemical activities. Thanks to iterative improvements, the process of using marketed drug structures as basis for repurposing, leads to increased safety and efficacy of therapeutic agents [41]. Our repurposing process started with finding similar chemical structured drugs with FDA approved drugs. First, we identify an original compound from FDA approved drugs with specific indications. Concerning the disease, we chose NSCLC as it occupied a large part of lung cancer [42]. 15 FDA approved drugs for NSCLC were selected from the human disease database (https://www.malacards.org/) as original compounds and compared with the misclassified antineoplastic drugs in our drug pool (S4 Table). To compute the similarity between two compounds, we utilized Library for the Enumeration of Modular Natural Structure (LEMONS) algorithm designed by Skinnider and Magarvey [25]. Tanimoto coefficient is identified as one of the best similarity matrixes, when estimating the similarities [43]. In this study, the drugs with tanimoto index above 0.8 were picked for further analysis (S5 Table). From 164 signaling pathways information of drugs embedded by Di-PASS, three pathways closely related to NSCLC therapies, including NSCLC pathway, epidermal growth factor receptor (EGFR) tyrosine kinase inhibitor resistance, and pathway in cancer were collected [44]. We listed all the drugs with negative Di-PASS scores in these three pathways from misclassified antineoplastic agents in S6 Table. Finally, the candidate drugs owning similar structures with FDA approved NSCLC drugs and negative Di-PASS score in NSCLC related pathways were obtained (Fig 5).

Take the first pair of drugs for example, pimozide was the repurposed potential drug produced by our pipeline. Gemcitabine is one of the most promising anti-cancer chemotherapy drugs, which has shown most activity in the treatment of NSCLC [45]. As an analogue, it is very possible for pimozide to share similar biological and physiochemical activities with gemcitabine. Not surprisingly, the pathway activation scores of pimozide showed -69.89, -13.83, and -20.76 in pathways of NSCLC, EGFR tyrosine kinase inhibitor resistance, and pathway in cancer, respectively. As mentioned previously, Di-PASS scores represented the intensity of pathway activating effects with positive/negative signs corresponding to activation/suppression. The high negative value of Di-PASS score indicates strong suppression effect of pimozide to NSCLC related pathways. Here an exciting potential drug candidate with strong evidences of both chemical structure and pathway signatures in new indication was discovered.

**Fig 6:**
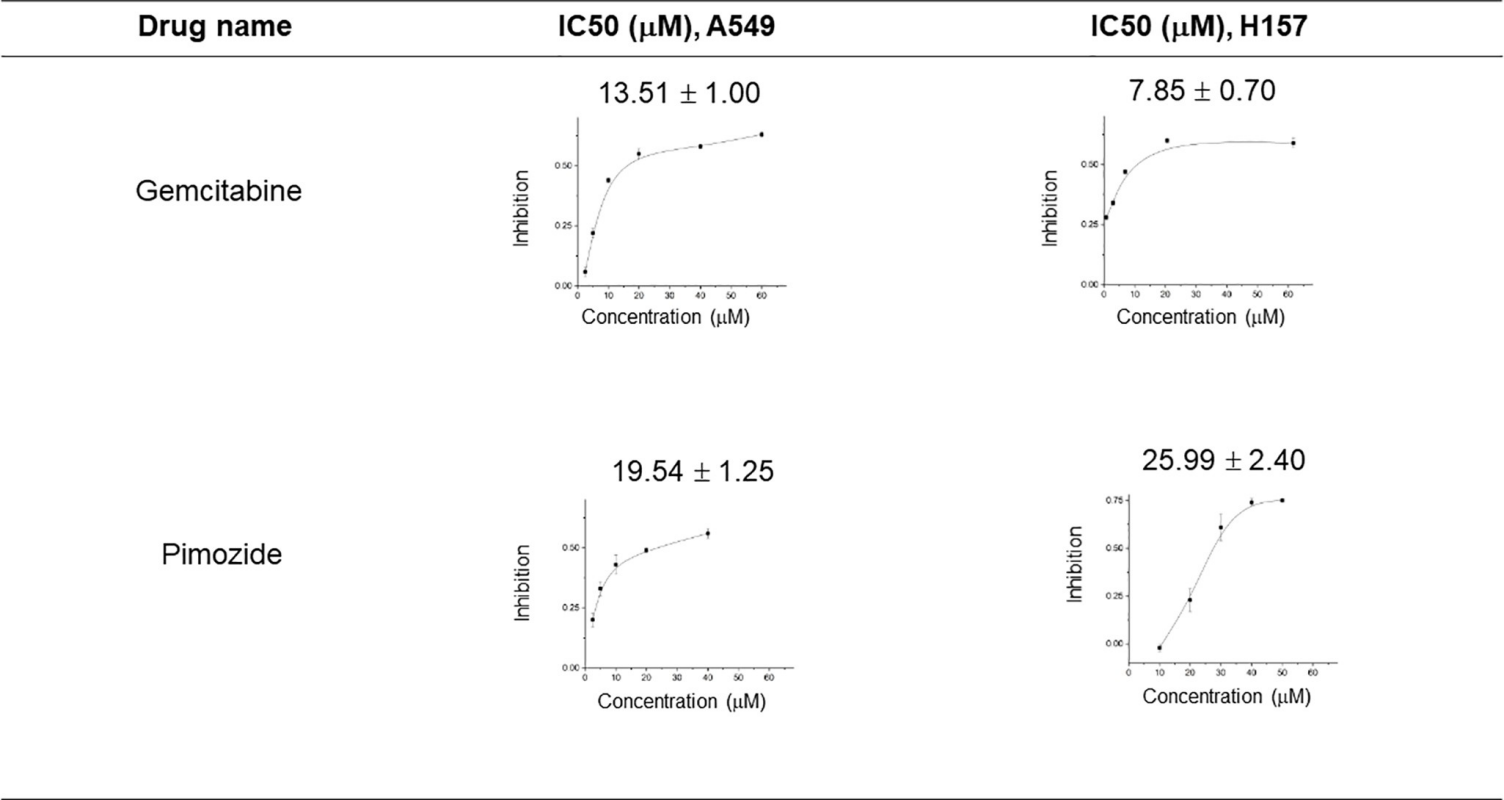
Cytotoxic activity of gemcitabine and pimozide on two NSCLC cell lines (A549 and H157). Test data expressed as the mean of three experiments.

To further validate the proposed candidate for repurposing, we did additional experiments in lab (Fig 6). Pimozide was evaluated using the sulforhodmide-B (SRB) assay [28] in two NSCLC cell lines (A549 and H157), and the antitumor activity was compared with the positive control (gemcitabine). These cell lines were chosen because they were characterized earlier for sensitivity to gemcitabine [46]. The *in vitro* results suggested that pimozide had comparable IC_50_ with gemcitabine and possibly warranted further evaluation in vivo. Both cell lines of A549 and H157 indicated that pimozide possessed activity for inhibiting NSCLC cells. The results were consistent with some earlier research based on pimozide [47, 48]. It is well known that pimozide is an anti-dyskinesia agent, which is used to reduce uncontrolled movements or outbursts of words caused by Tourette syndrome. However, we found that pimozide can be repurposed for treating NSCLC. Our study provided a pragmatic way to redirect traditional drugs to novel therapeutic uses. The other drug candidates were analyzed in the same way (S7 Table).

## Conclusions

We have proposed a novel drug repurposing approach based on transcriptomic data and chemical structures using DNN model. We have successfully repurposed pimozide from an anti-dyskinesia agent to an anti-NSCLC drug, which is supported by the *in vitro* experiments. The incorporation of both chemical structures and pathway activation scores when redirecting drugs to new therapeutic roles is proved to be an effective drug repurposing approach.

Although the Di-PASS algorithm can predict drugs with transcriptomic information (S8 Table provided entire drug list we can predict), it has been challenging to build assembled large-scale drug-transcriptomic datasets. In addition, further *in vitro* or *in vivo* experiments are still needed to warrant our predictions. Despite the above limitations, the approach can be extended to other diseases and drugs to identify novel therapeutic relationships.

## Supporting information

S1 Table Number of drugs selected for A375, HELA and HT29 cell lines according to MeSH therapeutic use section. Number of samples is shown in brackets.
(XLSX)

S2 Table MeSH category stratification binary matrix of 75 significantly perturbed drugs. Every drug belongs only to one category.
(XLSX)

S3 Table Misclassified drugs from antineoplastic agents. (TSV)

S4 Table FDA approved drugs of NSCLC from drug bank. (TSV)

S5 Table The drugs with tanimoto index above 0.8. (XLSX)

S6 Table The drugs with negative Di-PASS scores in three pathways related to NSCLC. (XLSX)

S7 Table Cytotoxic activity of potential drugs on two NSCLC cell lines (A549 and H157). SRB test data expressed as the mean of three experiments.
(XLSX)

S8 Table Drug list that Di-PASS can predict. (CSV)

S9 Table An input file example of Di-PASS. (CSV)

## Abbreviations

BGL: Boost Graph Library
BIRCH: Balanced Iterative Reducing Clustering using Hierarchies
Di-PASS: DIP^®^-Pathway Activation Scoring System
DNNs: Deep neural networks
EGFR: Epidermal growth factor receptor
FCFP: Functional-Class Fingerprint
FDA: U.S. Food & Drug Administration
GSEA: Gene Set Enrichment Analysis
HIF: Hypoxia-inducible factor
IC_50_: Half maximal inhibitory concentration
iPANDA: *in silico* Pathway Activation Network Decomposition Analysis
KEGG: Kyoto Encyclopedia of Genes and Genomes
LEMONS: Library for the Enumeration of MOdular Natural Structures
LINCS: Library of Integrated Network-Based Cellular Signatures
MeSH: Medical Subject Headings
NIH: National Institutes of Health
NSCLC: Non-small cell lung cancer
NTG: Nitroglycerin
OD: Optical density
OPTICS: Ordering Points To Identify the Clustering Structure
ReLU: Rectified linear unit
RF: Random forest
SRB: Sulforhodmide-B
